# Assessing the water quality impacts of two Category-5 hurricanes on St. Thomas, Virgin Islands

**DOI:** 10.1016/j.watres.2019.115440

**Published:** 2020-03-15

**Authors:** Sunny C. Jiang, Muyue Han, Srikiran Chandrasekaran, Yingcong Fang, Christina A. Kellogg

**Affiliations:** a Department of Civil and Environmental Engineering, University of California, Irvine, CA, USA; b St. Petersburg Coastal and Marine Science Center, U.S. Geological Survey, St. Petersburg, FL, USA

**Keywords:** Rain cistern, *Legionella*, Next generation sequencing, Disaster management, Virgin Islands

## Abstract

Managing waterborne and water-related diseases is one of the most critical factors in the aftermath of hurricane-induced natural disasters. The goal of the study was to identify water-quality impairments in order to set the priorities for post-hurricane relief and to guide future decisions on disaster preparation and relief administration. Field investigations were carried out on St. Thomas, U.S. Virgin Islands as soon as the disaster area became accessible after the back-to-back hurricane strikes by Irma and Maria in 2017. Water samples were collected from individual household rain cisterns, the coastal ocean, and street-surface runoffs for microbial concentration. The microbial community structure and the occurrence of potential human pathogens were investigated in samples using next generation sequencing. Loop mediated isothermal amplification was employed to detect fecal indicator bacteria, *Enterococcus faecalis*. The results showed both fecal indicator bacteria and *Legionella* genetic markers were prevalent but were low in concentration in the water samples. Among the 22 cistern samples, 86% were positive for *Legionella* and 82% for *Escherichia-Shigella*. *Enterococcus faecalis* was detected in over 68% of the rain cisterns and in 60% of the coastal waters (n = 20). Microbial community composition in coastal water samples was significantly different from cistern water and runoff water. Although identification at bacterial genus level is not direct evidence of human pathogens, our results suggest cistern water quality needs more organized attention for protection of human health, and that preparation and prevention measures should be taken before natural disasters strike.

## Highlights

• Rain cistern, coastal ocean and surface runoff waters were sampled post hurricanes.
• Microbial community composition was dramatically different in each type of water.
• Fecal indicator bacteria and *Legionella* were prevalent in all water samples.
• The concentrations of *Legionella* and fecal indicator bacteria were generally low.

## Graphical abstract

**Figure:**
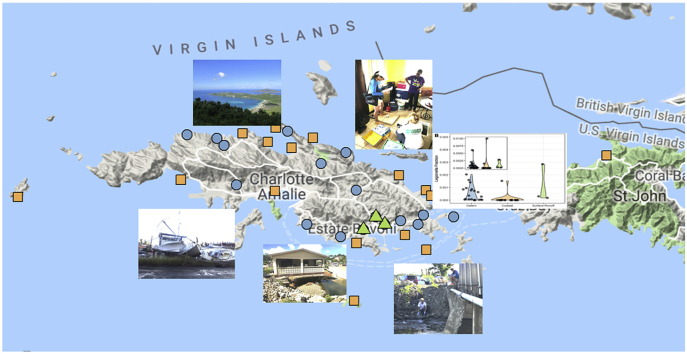

## 1 Introduction

The U.S. Virgin Islands (St. Thomas, St. John, and St. Croix) were devastated by back-to-back category-5 hurricanes, Irma and Maria, in the summer of 2017. Hurricane Irma, the strongest storm on record in the open Atlantic region, reached the Virgin Islands on September 6 (Cangialosi et al., 2018). The devastation can even be seen from space in NASA’s images: the green landscape images of St. Thomas were replaced by brown terrain (DeCosta-Klipa, 2017). Where Irma blew things down, Hurricane Maria, arriving 14 days later, washed them away. The massive flooding caused severe damage to the islands’ infrastructure. Roads were washed out; power and communication were down. With regional airports and shipping ports damaged, island residents were isolated from the mainland United States. Hurricane Maria’s impact on Puerto Rico further disrupted aid to the Virgin Islands. The approximately 100,000 people who live on these islands were largely on their own to find water, food, shelter and power immediately after the devastating storms.

On St. Thomas, rainwater collected in cisterns is the main source of household water. However, many of the collection systems were damaged during the hurricanes, leaving the quality of the water uncertain. Due to the shortage of electricity and disinfection chemicals, there was potential for little or no treatment of the rainwater. The Virgin Islands Water and Power Authority issued a boil water advisory for potable water customers territory-wide on Sept. 27, 2017 (WAPA, 2017). However, the lack of fuel on the island made the advisory impractical for the majority of the residents.

According to Virgin Islands Waste Management Authority, only approximately 30–35% of areas on the island are connected to the public sewer in St. Thomas, leaving the overwhelming majority of island properties to dispose of sewage through septic tanks or onsite wastewater treatment plants (Nowakowski, 2016). The lack of electricity paralyzed the sanitation systems that treat human sewage on the islands. Most of the septic systems were likely flooded by the stormwater, with raw human sewage possibly draining directly to surface water, coastal oceans and contaminating the rainwater cisterns. Fecal contamination of water resources is an imminent threat to human health with the likelihood of triggering large waterborne disease outbreaks. Even when bottled water replaces cistern water for drinking purposes, *Legionella*, the bacterial cause of Legionnaire’s disease, can be transmitted to humans through cistern water used for showering. Legionnaire’s disease is a common disease in the tropics, including documented epidemiological cases in the Virgin Islands (Schlech et al., 1985).

As part of the National Science Foundation’s RAPID research program on hurricane impacts, we conducted a field campaign to investigate water quality after the hurricanes struck St. Thomas. The goal of the study was to quantify the impact of hurricanes on water quality in order to develop strategies for preventive actions and effective post-storm management for human and environmental health protection. The field sampling campaign was carried out as soon as access to the island became feasible with the support of an ocean-going research vessel to transport lab supplies.

## 2 Materials and methods

**Fig. 1:**
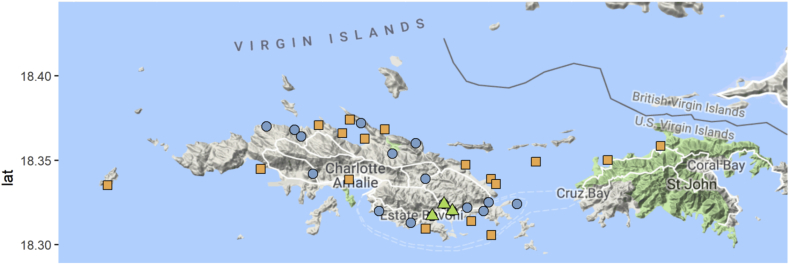
Water sample collection sites in St. Thomas, Virgin Islands. Blue circles indicate sites for rain cistern samples collection (n = 22), orange squares mark coastal sampling stations (n = 20) and green triangles indicate street surface runoff collection sites (n = 3). (For interpretation of the references to colour in this figure legend, the reader is referred to the Web version of this article.)

***Sampling sites and collection procedures*:** Field sampling campaigns were carried out November 20–26, 2017, nearly 3 months after the landfall of Hurricane Maria. A total of 22 water samples were collected from cisterns in residential households around the island (Fig. 1). Water temperature and salinity (in practical salinity units – PSU) in the cisterns were measured onsite using a ProPlus multimeter (YSI 605595). Water samples were collected directly from the top 1 m of each cistern using a peristaltic pump through bleach-sterilized tubing into a bleach-sterilized cubitaner (Fisher Scientific). The tubing and cubitaners were rinsed with the source water at least three times before 10 L of water sample were collected in the container and stored on ice in coolers for transport. The samples were filtered to capture microbial biomass using a temporary laboratory set up on a boat within 6 h of sample collection. Additional information about field conditions, sampling procedures and the locations of cisterns are presented in supplementary information (Table S1).

Coastal water samples from 20 locations around St. Thomas, and between the islands of St. Thomas and St. John, were collected using a 48 ft (14.6 m) powerboat that doubled as a field lab (Fig. 1). The exact sampling locations, conductivity and temperature at each site (Table S2) were recorded using a Castaway® CTD (SonTek). Water samples from the top 1 m of the ocean were pumped using the peristaltic pump as described for the cistern sample collection, but with the addition of an inline 0.8 μm prefilter (Avantec C080A142C) to remove plankton and larger particulates. Samples were filtered to capture microbial biomass onboard immediately after collection.

Three street surface-runoff samples were collected opportunistically (Table S3). The street surface-runoff samples were only collectable shortly after rainstorms due to the hilly topography of the island. Only running water on the streets was collected to avoid any confusion on the source of the water. The runoff was pooled by barriers and was pumped using the same peristaltic pump but with the addition of a mesh screen at the tubing intake as well as the 0.8 μm inline prefilter to reduce sand and gravel.

***Concentration of microbial biomass:*** Water filtration was carried out using a custom-made four-place filtration manifold with sterile 140 mL syringe barrels (Covidient 8881114063) as sample reservoirs connected to Sterivex-GP Pressure Filter Units (EMD Millipore SVGPL10RC). The syringe barrels were filled repeatedly with sample water until 5 L of water passed through the filter or until filtration time reached 1 h and 15min. For most coastal sites, 5 L of water sample passed through the Sterivex filter within 1 h (with the exception of three sites at the south side of the St. Thomas near the port of Charlotte Amalie). None of the 5-L cistern water samples was able to pass through the Sterivex filter units within 1 h. The filtration volume for these samples at the end of 1 h 15 min was recorded (Tables S1–S3). Further extending the filtration time only increased the filtration volume slightly indicating the filter was clogged. The filtration volume per unit time (1 h 15 min) is used to indicate the suspended solids and colloids in the water (similar to the concept of slit density index used in the water treatment industry) because a turbidity meter was not available during the field campaign.

After filtration, the Sterivex units were placed in sterile Whirlpak bags and flash frozen in liquid nitrogen immediately. The Sterivex units were kept in the liquid nitrogen dewar for storage during transport back to the mainland on the R/V *Walton Smith*. The frozen Sterivex units were shipped on dry ice from Florida to UC Irvine for sample processing.

***Microbial community analysis:*** At the UC Irvine lab, microbial biomass on Sterivex filters (stored at −80 °C freezer) was thawed and extracted using the DNeasy PowerWater Sterivex Kit (Qiagen 14600-50-NF) following the manufacturer recommended protocols. Aliquots of DNA extractions (20 *μ*L/sample, stored in 10 mM Tris) were shipped on dry ice to Molecular Research Lab (Shallowater, TX) for microbial diversity analysis using 16S rRNA gene V4 variable region. The sequencing was performed on an Ion Torrent PGM platform. Sequence data are available from NCBI’s Sequence Read Archive under Bioproject PRJNA554326.

Sequence data were processed using QIIME version 1.9.0 (Caporaso et al., 2010) following recommended settings for removal of barcodes, primers, short reads (<150bp), and reads with ambiguous base calls or with homo-polymers. USEARCH v10.0.240 (Edgar, 2013) was used for chimeric sequence removal. The UPARSE-OTU algorithm was used to define operational taxonomic unit (OTU, clustering with 97% similarity). Sample rarefaction was performed on the OTU table using the single_rarefaction.py function in QIIME. The sampling depth was determined based on the sample with the lowest number of sequencing reads. The final OTUs were taxonomically classified using SILVA 132 database with 97% identity (Quast et al., 2013).

Microbial diversity within each sample was assessed using Shannon index (Shannon, 1948), observed OTUs, Chao 1 index (Chao, 1984) and phylogenetic tree index. The between-sample diversities were compared across different types of samples using principal coordinate analysis (PCoA) performed on a matrix of weighted UniFrac distances (Paliy and Shankar, 2016). Indirect bootstrapped correlation analysis of PCoA values versus microbial OTU scores were used to estimate the contribution of each OTU to PCoA axes (Paliy and Shankar, 2016). The taxonomic profiles from different source waters were statistically compared using the Statistical Analysis of Metagenomic Profiles (STAMP) software package (Parks et al., 2014). Specifically, the abundance profiles (OTU table) produced by QIIME and a metadata file containing the descriptive information of each sample (including sampling location, salinity and temperature) were used as inputs. The statistical differences in mean proportion of taxa between sample groups were estimated with two-sided Welch’s *t*-test with 95% confidence intervals. *P*-value correction was performed using Benjamini-Hochberg false discovery rate (BH-FDR) approach (p < 0.05) as implemented in STAMP.

***Searching for potential human pathogens:*** The OTU table was searched using a list of potential human pathogens that have been identified in human sewage by metagenome analysis (Cai and Zhang, 2013). Due to the limitations of sequencing small fragments of the 16S rRNA gene, the taxonomic information is generally insufficient to identify bacteria to the species level (Janda et al., 2007). We focused on two bacterial genera in this report. *Escherichia-Shigella* is used as an indicator of fecal contamination. *Legionella* is used to indicate potential infection risks associated with aerosols since cistern water is more commonly used for showering than as a source of unfiltered drinking water. The fraction of *Legionella* and *Escherichia-Shigella* in each water sample was calculated by counting the number of OTUs associated with each genus. The relative abundance is calculated by the number of OTUs identified divided by the total OTUs in each sample. Other bacterial genera that may include potential human pathogens are presented in the Supplementary Table S4.

Factors that may contribute to the occurrence of *Legionella* and *Escherichia-Shigella* in samples were assessed using Pearson’s correlation coefficient in R (version 3.3.2). Input variables include phylogenetic diversity tree index, mean temperature and mean salinity of top 1m of coastal water or cistern water, and the top four principal coordinates from PCoA.

***Detection of Enterococcus faecalis*:** Loop mediated isothermal amplification (LAMP) was performed following the modified protocol of Kanitkar et al. (2017) to determine the occurrence of *Enterococcus faecalis* in aliquots of microbial DNA extracts from water samples. LAMP primers previously designed by Kato et al. (2007) targeting *azoA* gene of *E. faecalis* were adapted and are summarized in Table S5. The LAMP reaction was carried out in a total volume of 25 *μ*L containing 12.5 *μ*L of 1X WarmStart® LAMP Master Mix (New England Biolabs, MA, USA), 1.6 M each of primer FIP and BIP, 0.2 M each of primer F3 and B3, 0.8 M each of primer LB and LF, and 2 *μ*L of DNA extract. The negative control substituted 2 *μ*L of RNase-free water (QIAGEN, MD, USA) in the place of a DNA sample. The mixture was incubated at 65 °C for 30 min, and then cooled to 4 °C for 5 min to terminate the reaction. LAMP product was visualized by the addition of 1 *μ*L SYBR® Green I (1:10 dilution of 10,000X stock, Invitrogen™, Waltham, MA, USA). A subset of samples was diluted to assess the presence of amplification inhibitors. All diluted samples yielded negative LAMP results. Six replicate LAMP reactions were conducted for each water sample to calculate the most probable number (MPN) of genome concentration based on statistical probability. A numerical Excel spreadsheet was used for the estimation of MPN value with 95% confidence limits.

## 3 Results

***Water quality assessment by microbial diversity and composition:*** Comparison of 16S rRNA gene sequences from 45 water samples indicated that Proteobacteria were the most abundant phylum identified (Fig. S1). This result is not surprising because this phylum is composed of diverse families, genera and species that are commonly found in aquatic environments (Delmont et al., 2018; He et al., 2017). Bacteroidetes were the second most commonly observed phylum in all samples. Acidobacteria, Verrucomicrobia and Planctomycetes that were commonly found in cistern samples were rare in coastal and runoff waters (Fig. S1). Chlamydiae seemed to be unique to cistern and runoff water, and Gemmatimonadetes were in low abundance but ubiquitous in cistern water. Cyanobacteria and Marinimicrobia were present in all coastal samples but were absent in cistern samples. Cistern and runoff samples had more unidentified phyla than coastal waters (Fig. S1).

**Fig. 2:**
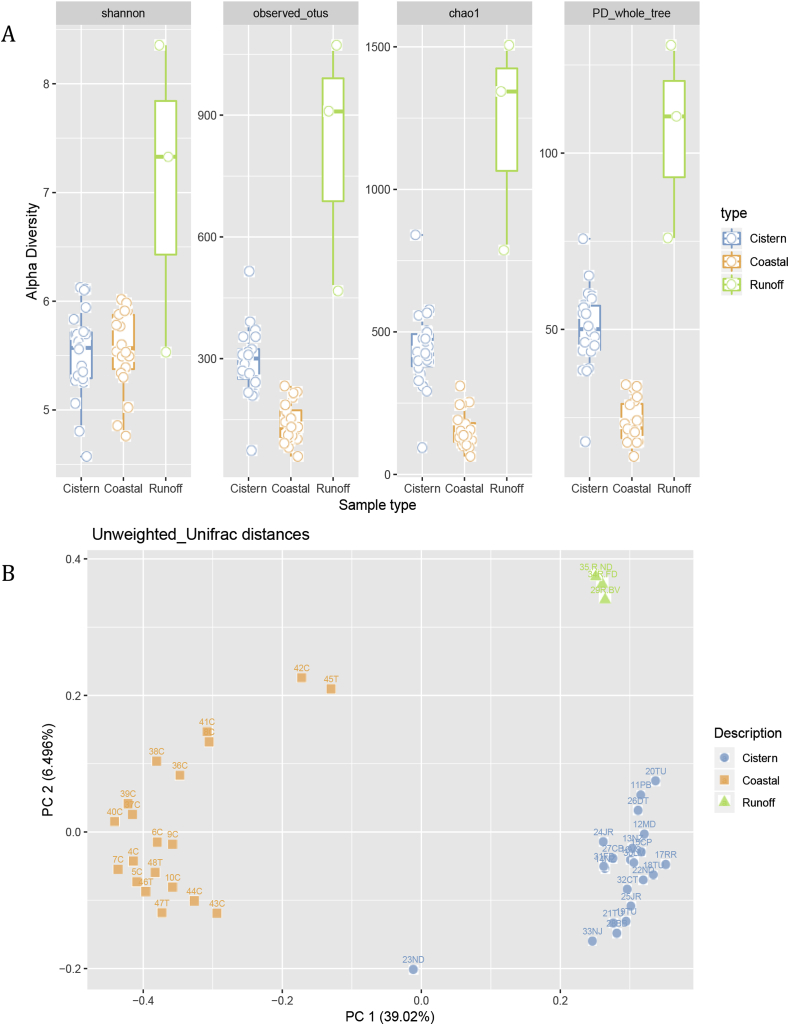
Alpha and beta diversity plot of microbial communities in water samples collected from coastal oceans, cisterns and street surface runoffs around St. Thomas, VI.

Within-sample phylogenic diversity indices are shown in Fig. 2A. Two sided t-tests showed that phylogenetic indices were significantly different between cistern and coastal samples (p = 10^−10^), and between coastal and runoff samples (p = 0.03). However, cistern and runoff samples were not significantly different (p = 0.07).

The PCoA plot showed that microbial communities were similar within the same type of samples but were different between sample types (Fig. 2B). Surface runoff samples were tightly clustered and were distantly related to other samples. Coastal samples had a greater variability as indicated by the spread along both principal coordinate 1 (PC1) and PC2 scales. Cistern samples were tightly clustered except sample 23ND, which had the lowest filtration-volume (860 ml), highest water temperature (28.2 °C) and salinity (0.15PSU)(Table S2). The distinct microbial community composition in this sample together with physical water quality parameters implied contamination of the cistern by flood waters (corroborated by the home owner’s description of the storm events).

**Fig. 3:**
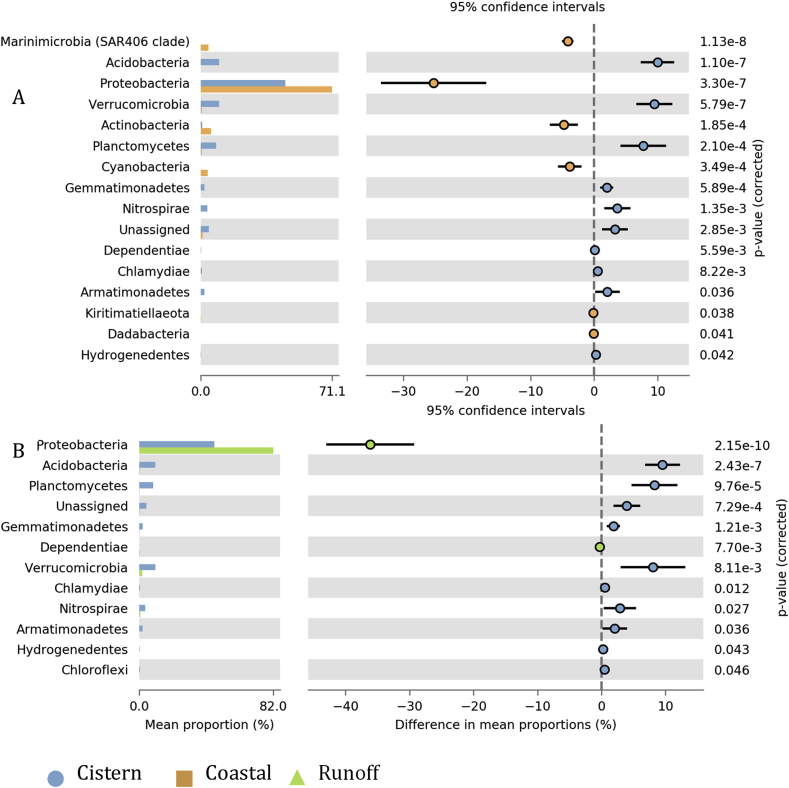
Statistical comparison of microbial composition of samples collected from rain cisterns verses coastal oceans (A), and rain cisterns verse surface runoffs (B) at phylum level.

Statistical comparison of microbial community composition in coastal waters versus those in cistern waters revealed that Marinimicrobia, Acidobacteria, Proteobacteria and Verrucomicrobia were the main phyla that contributed to the observed differences (Fig. 3A). Differences in Proteobacteria among cistern and runoff samples determined their unique microbial signature (Fig. 3B).

***Occurrence of Escherichia-Shigella* and *Legionella***: *Escherichia-Shigella* were frequently found in cistern waters, with 82% of the samples containing gene sequences matching these genera (Table 1). Thirty percent (30%) of the coastal water samples also contained 16S rRNA gene sequences that identified as *Escherichia-Shigella*. One of three street surface-runoff samples had a match with *Escherichia-Shigella* gene sequences but all three surface-runoff samples were positive for *Legionella*. *Legionella* was also detected in 86% of rain cisterns and 10% of coastal waters (Table 1).

**Table 1:** Occurrence of pathogenic genera and fecal indicator in water samples.

Pathogenic genera or fecal indicator	Sample Types (% positive)		
	Cistern (n = 22)	Coastal (n = 20)	Surface Runoff (n = 3)
Escherichia-Shigella	82%	30%	33%
Legionella	86%	10%	100%
*Enterococcus faecalis*	68%	60%	67%

**Fig. 4:**
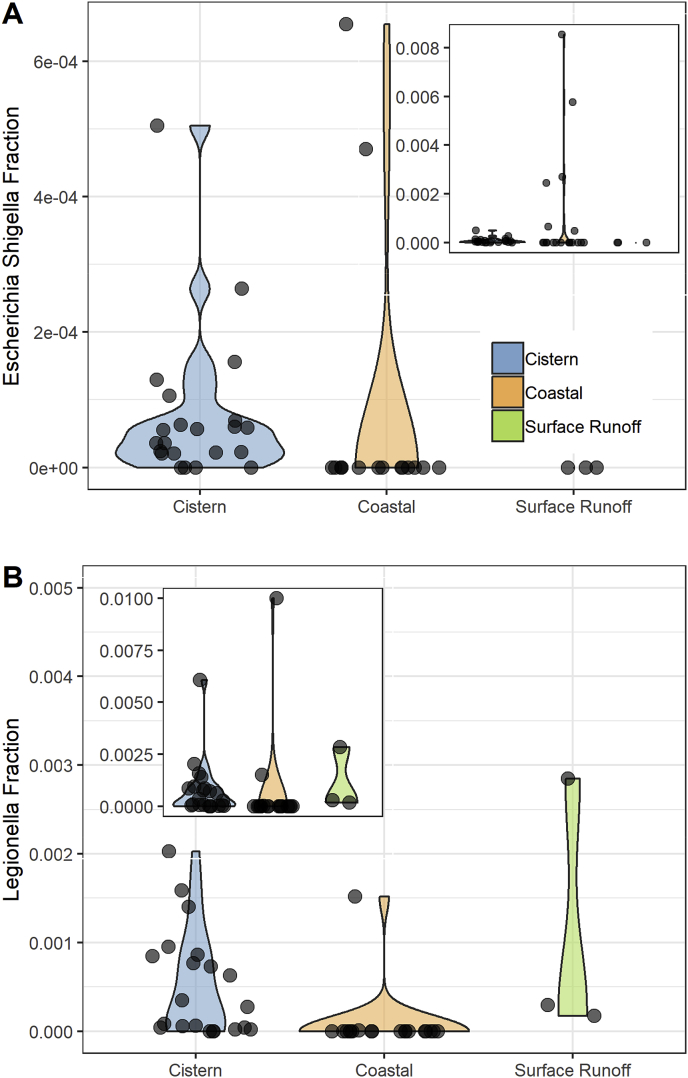
Fraction of *Escherichia-Shigella* (A) and *Legionella* (B) genera identified in different source waters. The width of the plot indicates the number of samples in a region. The small inserts show all the samples with extended y-axis.

The relative abundance of OTUs identified as *Escherichia-Shigella* and *Legionella* was plotted by sample types (Fig. 4). The occurrence of these genera as a portion of the total microbial population varied largely among samples, ranging from non-detect to 0.8% *Escherichia-Shigella* (Figs. 4A) and 1% of *Legionella* (Fig. 4B) in a coastal sample. The two genera, although frequently detected in cistern waters, accounted for very small fractions of the total microbial population (<0.05% for *Escherichia-Shigella,* <0.4% for *Legionella*). The majority of the cistern samples had less than 0.1% of the two genera combined as indicated by the width of the plot at the base (Fig. 4A and B). The fractions of these genera were even lower in most of coastal waters with exception of a couple heavily polluted samples from south side of the island (see small insert in Fig. 4A and B).

**Fig. 5:**
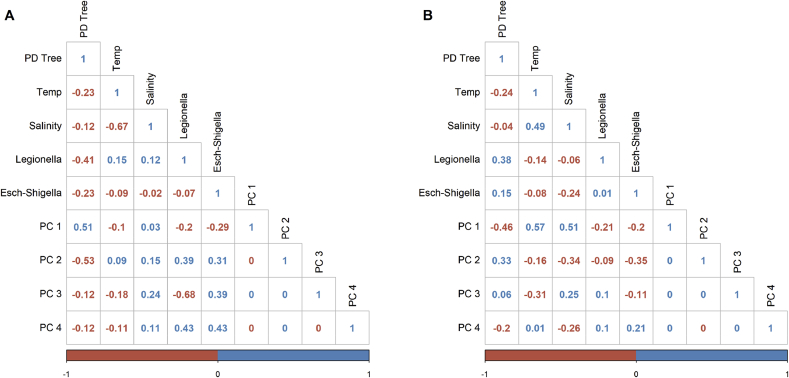
Pearson correlation analysis of water quality parameters, phylogenetic diversity, and occurrence of pathogenic bacterial genera. A) Coastal ocean samples; B) Rain cistern samples.

Results of Pearson correlation analysis of water quality parameters, phylogenetic diversity (PD Tree index), PCoA coordinates, and fractions of *Escherichia-Shigella* and *Legionella* genera in each sample are shown in Fig. 5. There was a significant negative correlation between water temperature and salinity at coastal sites since lower salinity waters were found at coastal zones that were heavily influenced by land runoff (Fig. 5A). The fraction of *Legionella* detected in coastal samples was found to be negatively correlated with PC3 of the PCoA (Fig. 5A), while the fraction of *Escherichia-Shigella* genus had a positive correlation with PC3, suggesting these two genera may originate from different sources. The detection of both genera in coastal samples was negatively correlated with microbial diversity index, indicating they were likely found in samples with lower microbial diversity. There are no significant correlations between the measured environmental parameters, microbial diversity and detection of *Escherichia-Shigella* and *Legionella* genera in cistern waters (Fig. 5B). This suggests additional factors that were not measured in our study determine the occurrence of these two genera.

**Fig. 6:**
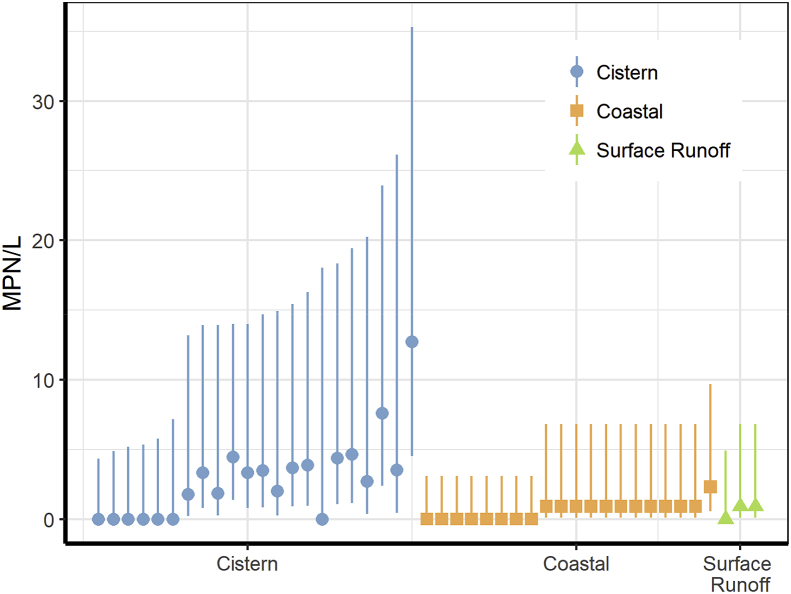
Detection of *E. faecalis* by MPN-LAMP assay in water samples. The dots indicate MPN and the vertical line on each dot indicates confidence intervals for each sample.

***Detection of fecal indicator bacteria:*** *E. faecalis* is a commonly used indicator of fecal pollution in water. LAMP assay results showed positive detection of *E. faecalis* in 68% of the cistern water samples, 60% of coastal waters and 67% of street surface runoff (Table 1). The concentrations of *E. faecalis* were generally low as shown in Fig. 6 but had large confidence intervals due to the lower statistical power of the data. The higher MPNs were found in cistern water samples in comparison with coastal waters and runoff water samples.

## 4 Discussion

Water quality degradation and related waterborne disease outbreaks are major concerns in the aftermath of natural disasters. However, water quality data in disaster regions are limited due to the difficulty of carrying out field investigations while other immediate rescue efforts take priority. This is especially the case for oceanic islands, where access to the region can be cut off due to paralyzed transportation systems. The Virgin Islands are an American territory that heavily relies on the tourism industry for the local economy. However, 22 percent of the population in the Virgin Islands lives in poverty (2010 U.S census). Fifty percent of those living below the poverty level were families led by single mothers (Jung, 2017). Poverty and poor health condition go hand in hand as evidenced by the frequent reports of infectious disease outbreaks in developing nations. The study presented here is one of few studies of water quality in disaster regions, and perhaps the only systematic investigation of water quality issues in a remote tropical island post natural disasters.

The results of this study revealed that fecal indicator bacteria were prevalent in cistern waters that were used for household water supplies. *Legionella*, the major causative agent of respiratory infections through shower water, was detected in over 86% of cisterns. Although not all species within the genus are pathogenic, the high frequency of detection of this genus brings attention to the proper management of cistern water. Future work should screen cistern water specifically for genetic markers of pathogenic species of *Legionella* in order to develop effective remediation strategies in St. Thomas.

Biofilms in cisterns could be a source of the pathogen as suggested by previous work showing biofilms in water distribution systems as the source of *Legionella* bacteria (Abu Khweek and Amer, 2018). *Legionella* is also ubiquitous in natural environments including soil and leaf litters (van Heijnsbergen et al., 2015). Floodwater that mobilizes surface soil may also serve as the carrier to bring the pathogen into cisterns. This is supported by the positive detection of *Legionella* in all three street-runoff samples. Birds may also be a vector (Santos et al., 2012). What is not clear in the results of this study is the degree of impact of hurricanes on cistern water quality since the privately-owned cisterns are not routinely monitored for water quality. There is a lack of historical data of cistern water quality that can be used as a baseline. The only studies conducted in Virgin Islands cisterns were from over 30 years ago, in which both fecal bacteria and *Legionella* were reported (Broadhead et al., 1988)(Crabtree et al., 1996). Outbreaks of Legionnaire’s disease have also been documented in 1985 in St. Thomas (Schlech et al., 1985).

Regardless of impact of the hurricanes on cistern water quality, the outcome of this study suggests cistern water quality needs more organized attention. Leaving residents of low socioeconomic status to manage their own water supply creates gaps in the National Safe Drinking Water Act protection from waterborne diseases. Reported cases of waterborne diseases, as have been shown by recent focused effort, are only the tip of the iceberg (Yang et al., 2012). The Federal and island governments should not wait until a major outbreak strikes the community to act on water quality assurance. Distribution of bottled water for drinking as part of post-disaster management can help to prevent water ingestion-related waterborne diseases, but respiratory illnesses that are acquired through shower water may become a bigger outbreak threat. Preparation and prevention measures (i.e. cistern water management) should be taken before natural disasters strike. There is a need for routine cistern water quality monitoring to demonstrate background concentrations of potential pathogens, which then allows evaluation of storm event influences and guides bioremediation efforts.

The impact of hurricanes and floodwaters on coastal water quality degradation is easier to see in comparison with that on cistern waters. Fecal indicator bacteria (*Escherichia-Shigella* and *Enterococcus faecalis*) were found in coastal water, especially near the southeast end of the island where land runoff had a significant impact on water quality. Although they make up small fractions of the total microbial community, there was a clear sign of coastal water pollution. Since tourism is a mainstay of the island economy, the degradation of recreational water quality not only impacts human health but also the local economy.

From a fundamental research point of view, clear differences in microbial community structure were found in the three different types of water samples analyzed. Microbial diversity and community structure may be viewed as a signature of environmental conditions at a specific location and time. Such signatures, although not currently used as a quantitative index of water quality measures, may become metadata for comparison of environmental perturbation and for discovery of water quality changes with the continuous enrichment of data (due to reduced costs of sequencing) and the rapid advancement of data science. The capability of next generation sequencing to categorize water quality, including detection of genera containing potential pathogens in a microbial community, can offer a water quality assessment tool for water management. Moreover, although the study was not designed to assess the ecological burden from hurricanes to marine environments surrounding the island (e.g., mangroves, seagrass beds, coral reefs), the microbial diversity data might be useful for such a purpose (Bruce et al., 2012).

The major drawback of genetic based analytical methods is their inability to distinguish active bacteria from residual genetic materials. One may argue the detection of *Legionella* and the prevalence of the *Escherichia-Shigella* markers are not good representations of their infectivity and health risk. Studies in wastewater treatment plants have shown that disinfected wastewater had similar genomic copies of bacterial target but low cultivable fecal bacteria, suggesting genetic methods can significantly overestimate target bacteria (He and Jiang, 2005). This may well be the case in cistern waters, given that residents understand that animal feces (e.g., from birds) from the roof are an expected ‘bycatch’ with rainwater, leading to regular additions of bleach to cistern water for disinfection. Viability dyes such as ethidium monoazide (EMA) and propidium monoazide (PMA) have been used in conjunction with qPCR and Illumina next generation sequencing platforms - this could provide an indication of viable and intact cells (Reyneke et al., 2017) in future research. But until then, the detection of genetic markers, at minimum, represents the recent contamination by potential pathogens. MPN-LAMP provided the best estimates of concentration of *Enterococcus* in the absence of the conditions for culture assay in the post-disaster area.

In comparison with PCR, LAMP has improved specificity due to the adoption of six sets of primers (Martzy et al., 2017). In comparison with a quantitative PCR assay, the LAMP reaction is more tolerant to the presence of inhibitors. Similar to PCR, the LAMP assay has better sensitivity than using short fragments of the 16S rRNA gene for identification of specific species (Cai and Zhang, 2013). In fact, *Enterococcus* spp. were not identified in our OTU table. The LAMP assay identified a low level of these fecal indicator bacteria in surface runoff and coastal waters in comparison to the recreational water quality criteria (US EPA, n.d.). The large volume of stormwater flow following the hurricanes could dilute the fecal pollution.

To better understand the risk from exposure to poor water quality, a quantitative risk assessment should be carried out to connect the exposure model with pathogen concentrations in the water. Since the majority of people received the government-issued water quality warning and adopted bottled water as their main source of drinking water (based on surveying cistern owners), the risk of gastroenteritis from fecal pathogens in cistern waters is likely low. Respiratory illness from shower water exposure, however, is of greater concern. The health effects could be exacerbated by the mental stress and anxiety experienced by disaster-impacted local residents. A future study of health effects will be helpful to better understand the water quality and health risk in the aftermath of disasters.

## 5 Conclusions

A field sampling campaign for water quality assessments on a hurricane-impacted tropical island were carried out. The results of the study showed:

1. Fecal indicator bacteria and *Legionella* were prevalent in all water samples
2. Microbial community structures in water collected from cisterns, coastal oceans and street surface runoff were dramatically different
3. The concentrations of *Legionella* and fecal indicator bacteria were generally low
4. In spite of the lack of infectivity data on *Legionella*, the prevalence of this genetic marker is of great concern due to the likelihood of its transmission through shower water

## Declaration of competing interest

The authors declare that they have no known competing financial interests or personal relationships that could have appeared to influence the work reported in this paper.
