# Supplementary material for: Assessing the water quality impacts of two Category-5 hurricanes on St. Thomas, Virgin Islands
Source: Water Res. 2020 Mar 15;171:115440. doi: 10.1016/j.watres.2019.115440 (PMC7001102; doi:10.1016/j.watres.2019.115440)
Supplement: Multimedia component 1 [file mmc1.docx]

Assessing the Water Quality Impacts of Two Category-5 Hurricanes on St. Thomas Virgin Islands

Sunny C. Jiang^1*^, Muyue Han^1**^, Srikiran Chandrasekaran^1^, Yingcong Fang^1^, Christina A. Kellogg^2^

^1^Department of Civil and Environmental Engineering, University of California, Irvine, CA

^2^St. Petersburg Coastal and Marine Science Center, U.S. Geological Survey, St. Petersburg, FL

*Corresponding author, 844 Engineering Tower, Irvine, CA 92797; [sjiang@uci.edu](mailto:sjiang@uci.edu)

**Current affiliation, University of Illinois at Chicago

Supplemental Information

**Additional information for sampling condition, sites and collection procedures:** The research team reached St. Thomas on November 16^th^ on one of the few commercial flights from mainland to the island, nearly 3 months after the landfall of Hurricane Maria. The sampling supplies and liquid nitrogen were transported by the R/V *Walton Smith* from Miami to St. Thomas. Field sampling campaigns were carried out November 20–26, 2017. A temporary laboratory for water filtration was set up on the aft deck of a 48 ft powerboat that survived the storm. A gasoline generator was used as the power source for water filtration. A total of 24 water samples were collected from cisterns in residential households around the island (Figure 1). The cisterns were sampled through access hatches, which were located either inside or outside the homes. Water temperature and conductivity in the cistern were measured onsite using a ProPlus multimeter (YSI 605595). Water samples were collected directly from the top 1 meter of each cistern using a peristaltic pump through bleach-sterilized tubing into a bleach-sterilized cubitaner (Fisher Scientific). The tubing and cubitaners were rinsed with approximately 0.5 liters of water sample each time for at least three times, before 10 liters of water sample was collected into the container and stored in iced coolers for transport. The samples were concentrated for microbial biomass at the temporary laboratory within 6 hours of sample collection.

**Table S1. Water quality parameters collected in cisterns at different residential areas of St. Thomas, VI**

| Sample ID | Residential Area | Temp (°C) | Salinity (PSU) | | DO (mg/L) | *Filtration Vol. (ml) |
| --- | --- | --- | --- | --- | --- | --- |
| 11PB | Peterborg | 26.2 | 0.08 | | NA | 3180 |
| 12MD | Mandal | 25.9 | 0.03 | | NA | 2300 |
| 13NZ | Nazareth | 26.4 | 0.04 | | NA | 3540 |
| 14NZ | Nazareth | 27.3 | 0.14 | | NA | 2910 |
| 15CP | Cabrita Point | 27.2 | 0.12 | | NA | 2160 |
| 16VS | Vessup Bay | 27.2 | 0.05 | | NA | 2290 |
| 17RR | Ridge Road | 27 | 0.09 | | NA | 2160 |
| 18TU | Tutu | 27.3 | 0.04 | | NA | 2680 |
| 19TU | Tutu | 27.2 | 0.03 | | NA | 3930 |
| 20TU | Tutu | 26.6 | 0.12 | | NA | 1680 |
| 21TU | Tutu | 27.7 | 0.06 | | NA | 2860 |
| 22ND | Nadire | 27.8 | 0.06 | | NA | 3000 |
| 23ND | Nadire | 28.2 | 0.15 | | NA | 860 |
| 24JR | St. Joseph & Rosendal | 26.9 | 0.08 | | NA | 1300 |
| 25JR | St. Joseph & Rosendal | 25 | 0.04 | | NA | 2720 |
| 26DT | Est. Dorothea | 24.3 | 0.04 | | NA | 3580 |
| 27CB | Upper Caret Bay (Side of Crown Mountain) | 24.1 | 0.03 | | 4.3 | 2580 |
| 28BB | Bolongo Bay | 27.1 | 0.03 | | NA | 3020 |
| 30LM | Estate Longmath | 26.7 | 0.08 | | 6.4 | 2280 |
| 31FB | Frenchman's Bay | 26.6 | 0.04 | | NA | 2440 |
| 32CT | Contant | 26.3 | 0.04 | NA | | 3000 |
| 33NJ | Neltjeberg | 25.1 | 0.03 | 5.8 | | 2580 |

*Filtration volume recorded after 1 hour 15 mins of filtration (it is used to indicate the suspended solids and colloids content in the water)

**Table S2. Water quality parameters collected from coastal sampling sites around St. Thomas, VI.**

| Sample ID | Location and Coordinates | Temp* (°C) | Salinity* (PSU) | Density* (kg/m^3^) | Specific conductance* (µS/cm) |
| --- | --- | --- | --- | --- | --- |
| 4C | Buck Island (18.277, -64.889) | 28.85 | 34.44 | 1021.70 | 52297.97 |
| 5C | Bovoni Landfill offshore (18.309, -64.888) | 29.06 | 34.32 | 1021.54 | 52137.14 |
| 6C | Charlotte Amalie (18.339, -64.934) | 29.49 | 33.43 | 1020.72 | 50924.62 |
| 7C | Black Point (18.345, -64.987) | 29.59 | 34.08 | 1021.18 | 51811.32 |
| 8C | Coculus Rock (18.314, -64.86) | 29.26 | 33.11 | 1020.56 | 50494.95 |
| 9C | Coculus Rock (offshore) (18.306, -64.848) | 28.81 | 34.28 | 1021.59 | 52079.05 |
| 10C | Sapphire Beach (18.339, -64.849) | 28.56 | 34.78 | 1022.05 | 52754.10 |
| 36C | Water Bay (18.347, -64.864) | 28.33 | 34.15 | 1021.65 | 51898.58 |
| 37C | Coki Beach (18.351, -64.867) | 28.52 | 34.79 | 1022.07 | 52763.37 |
| 38C | Off Mahogany Run (18.368, -64.913) | 28.50 | 34.81 | 1022.09 | 52794.01 |
| 39C | Magens Bay 1 (18.374, -64.934) | 28.53 | 34.83 | 1022.10 | 52820.27 |
| 40C | Magens Bay 2 (West) (18.366, -64.938) | 28.47 | 34.21 | 1021.66 | 51987.03 |
| 41C | Magens Bay 3 (Beach) (18.363, -64.925) | 28.53 | 34.87 | 1022.13 | 52873.07 |
| 42C | Hull Bay (18.371, -64.953) | 28.69 | 34.81 | 1022.03 | 52788.43 |
| 43C | Botany Bay (18.36, -65.034) | 28.94 | 34.69 | 1021.86 | 52635.73 |
| 44C | Savanna Island (18.335, -65.08) | 28.85 | 34.50 | 1021.74 | 52369.97 |
| 45T | Sapphire Beach (18.336, -64.846) | 28.55 | 34.62 | 1021.93 | 52533.50 |
| 46T | Pillsbury Sound (18.349, -64.822) | 28.60 | 34.56 | 1021.87 | 52456.33 |
| 47T | Hawksnest Bay (18.35, -64.778) | 28.56 | 34.55 | 1021.87 | 52435.81 |
| 48T | Maho Bay (18.358, -64.746) | 28.63 | 34.81 | 1022.05 | 52793.75 |

*Measurement reported as average over 1m of CTD probe.

**Table S3. Water quality parameters collected in street surface runoffs on St. Thomas, VI**

| Sample ID | Residential Area | Temp (°C) | Salinity (PSU) | DO | *Filtration Vol. (ml) |
| --- | --- | --- | --- | --- | --- |
| 34R-FD | Estate Frydenhoj | 30.7 | 0.93 | NA | 5000 |
| 35-R-ND | Nadir (Under Bridge) | 28.9 | 0.78 | 9.05 | 5000 |
| 29 R-BV | Runoff Bovoni | 30.5 | 0.69 | NA | 3160 |

*Filtration volume recorded after 1 hour 15 mins of filtration if 5 liters of water did not pass through in 1 hour.

**Table S4. Potential pathogenic genera searched* and identified among OTUs.**

| Bacterial Genera | Cistern | Coastal | Runoff |
| --- | --- | --- | --- |
|  | **% Positive samples** | | |
| *Corynebacterium* | 14% | 40% | 0% |
| *Corynebacterium 1* | 14% | 45% | 0% |
| *Campylobacter* | 0% | 0% | 0% |
| *Bacillus* | 77% | 30% | 67% |
| *Citrobacter* | 86% | 15% | 67% |
| *Enterocuccus* | 0% | 0% | 0% |
| *Escherichia-Shigella* | 82% | 30% | 33% |
| *Legionella* | 86% | 10% | 100% |
| *Listeria* | 0% | 0% | 0% |
| *Mycobacterium* | 95% | 0% | 33% |
| *Neisseria* | 0% | 5% | 0% |
| *Pseudomonas* | 100% | 95% | 100% |
| *Salmonella* | 0% | 0% | 0% |
| *Staphylococcus* | 59% | 45% | 33% |
| *Mycoplasma* | 0% | 15% | 33% |
| *Photobacterium* | 0% | 15% | 0% |
| *Vibrio* | 5% | 30% | 100% |

*List of potential waterborne human pathogenic genera from the reference of Cai et al. 2013 was used in the search.

Reference cited:

Cai, Lin, and Tong Zhang. "Detecting human bacterial pathogens in wastewater treatment plants by a high-throughput shotgun sequencing technique." *Environmental science & technology* 47.10 (2013): 5433-5441.

**Table S-5*. Oligonucleotide Primers for Enterococcus Faecalis LAMP assay***

| Primer | Sequence |
| --- | --- |
| F3 | 5’-GCCGGAAATCGATGAAGA-3’ |
| B3 | 5’-TCCAGCAACGTTGATTGT-3’ |
| FIP | 5’-CACTTTTTGTTGTTGGTTTTCGCTTTATTATCTGCTTGGGGTGC-3’ |
| BIP | 5’-ATCTGCAGACAAAGTAGTAATTGCTCCAAGCTTTTAAGCGTGTC-3’ |
| LoopF | 5’-AAATGCTGCGCCAGCTCG-3’ |
| LoopB | 5’-TCCAATGTGGAACTTAAACGTACC-3’ |

**Supplemental Figure:**


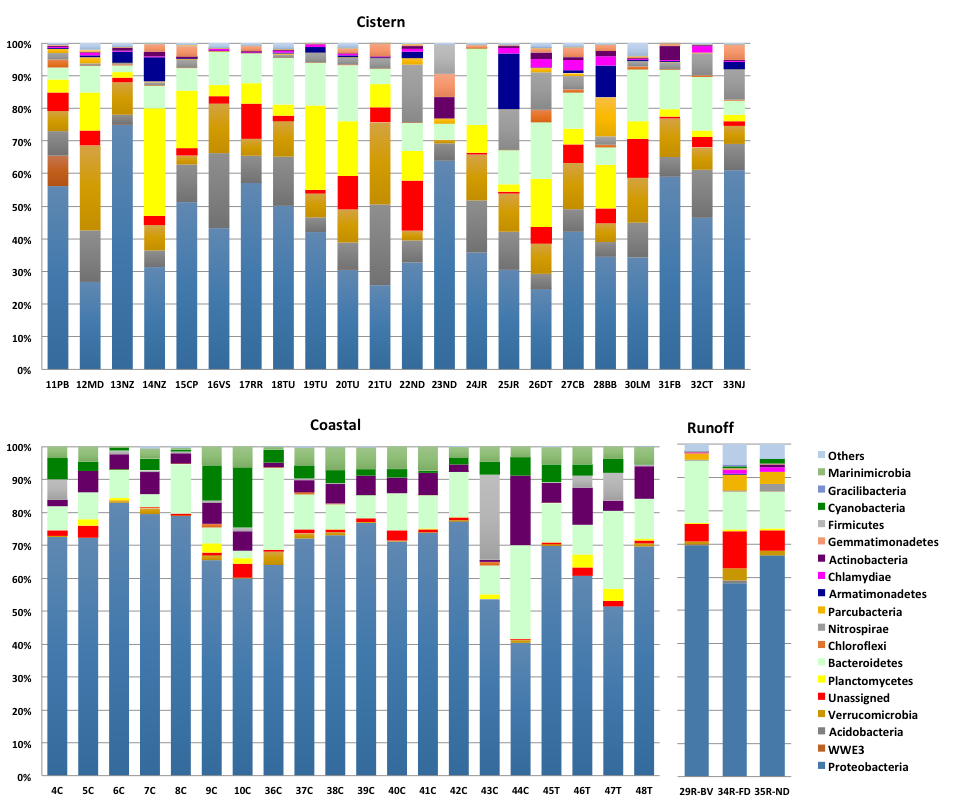


Figure S1. Top 19 phyla identified by 16S rRNA gene sequences among samples collected from rainwater cisterns, coastal ocean near St. Thomas and street surface runoff.
